# Draft whole-genome sequence of *Enterococcus faecium* isolate from a clinical sample from Dhaka, Bangladesh

**DOI:** 10.1128/mra.01195-24

**Published:** 2024-12-31

**Authors:** Syeda Tasneem Towhid, Md Maksudur Rahman Shihab, Mohammad Jubair, Naimul Islam Faisal, Md. Mustafizur Rahman

**Affiliations:** 1 Department of Microbiology, Jagannath University 421982 , Dhaka, Bangladesh; 2 Department of Oncological Science, University of Utah, Salt Lake City, Utah, USA; 3 Infectious Disease Division, International Center for Diarrheal Disease Research, Dhaka, Bangladesh; Queens College Department of Biology, Queens, New York, USA

**Keywords:** *Enterococcus*, genome analysis, Dhaka, Bangladesh

## Abstract

We collected a urine specimen from a 20-year-old male, cultured it on Chrom-agar *Enterococcus*, identified it with VITEK2, and whole-genome sequenced it using the PacBio RSII. The genome was 3,111,621 nucleotides long with a GC% of 37.6%. It was identified as a human pathogen with a 92.3% probability and is available on NCBI with an accession ID of JBFBPS000000000.1.

## ANNOUNCEMENT

The Enterococci isolates from Bangladesh do not produce amplicons with *ddl* primers or *sod* primers described in the literature (1). Signature primers must be available for the detection of pathogens with a cost-effective technique (conventional PCR) to study many samples. Therefore, we sequenced a local vancomycin-resistant *Enterococcus faecium* isolate from Dhaka, Bangladesh, so that signature primers can be designed for identification during surveillance. The sample was collected on 18 May 2018 (coordinates 23.7450° N, 90.3760° E) from the urine of a 20-year male with an acute urine infection, who was otherwise healthy. The urine specimen was first cultured on Chrom Agar *Enterococcus* and then detected with VITEK2 (ID 130002045353671) with 97% similarity. This isolate is sensitive to 10 µg penicillin, 5 µg ciprofloxacin, 10 µg erythromycin, and 30 µg tetracycline, intermediate sensitive to 10 µg gentamycin, 30 µg teicoplanin, and 15 µg tigecycline, and resistant to 30 µg vancomycin. The DNA purification protocol followed a modified version of the CDC PULSENET Total DNA Extraction Protocol (2), employing the Qiagen DNeasy Blood & Tissue Kit (Qiagen, 69504) for efficient purification of bacterial DNA. After evaluating the extracted DNA using both Nanodrop (Thermo Fisher Scientific, ND-1000) and Qubit 4.0 (Thermo Fisher Scientific, Q33238) measurements, the PacBio DNA Sequencing Kit, which includes reagents specifically for the preparation of SMRT (Single Molecule, Real-Time), was utilized for library preparation. The prepared DNA libraries were subjected to sequencing using the PacBio RSII platform. Sequencing was carried out with paired-end 2 × 150 bp reads, yielding comprehensive genetic information.

### Whole genome sequence analysis

The data underwent a trimming process to remove low-quality sequences, employing tools such as BBDuk version 39.01 (https://github.com/BioInfoTools/BBMap) and Fastp version 0.23.4 (https://github.com/OpenGene/fastp). The high-quality, trimmed reads were then ready for subsequent analysis steps.

The trimmed reads were assembled using the SPAdes version 3.15.5 (3, 4) algorithm, which constructed scaffolds from the sequence data. Following assembly, the scaffolds were annotated using the Prokka version 1.14.5 (https://github.com/tseemann/prokka) annotation tool, and the genome was analyzed by AMRFinder version 3.11 (https://ftp.ncbi.nlm.nih.gov/pathogen/Antimicrobial_resistance/AMRFinderPlus/database/), which identified *ant* (6)-Ia and *aph*(3′)-III genes responsible for resistance against gentamicin, tobramycin, and tetracycline, *cfr*(D), *crmA,* and *OptrA* rendering resistance against chloramphenicol and glycopeptides. The VanH AX_z gene was the major contributor to resistance against vancomycin and teicoplanin (5, 6).

For comparative genomic analysis, tools such as GAMMA version 2.2 (https://github.com/rastanton/GAMMA) and Kraken2 version 2.1.2 (https://github.com/DerrickWood/kraken2/wiki/Manual) facilitated the comparison of our genomic data against curated databases. Analysis of the assembled contig with Pathogenfinder tool v2 and Virulencefinder tool v1 showed the isolate had a 92.3% probability of being a human pathogen; the major virulence factors were collagen adhesion protein (ACM), enterococcal surface protein (esp), and *Enterococcus faecalis* endocarditis-associated virulence factor A (efaA) (7 ,8).

This sequence is available on NCBI with an accession ID of JBFBPS000000000.1.

## Data Availability

The raw reads can be found at NCBI Sequence Read Archive (SRA), accession number SRP536898. The assembly of raw reads is available at NCBI BioProject, accession number PRJNA1132561 and BioSample accession number SAMN42349775. The complete genome sequence is available in GenBank under accession number JBFBPS000000000.1.
